# Gelatin-Modified Bioactive Glass for Treatment of Dentin Hypersensitivity

**DOI:** 10.3390/ijms252211867

**Published:** 2024-11-05

**Authors:** Mengzhen Tang, Min Ge, Xu Zhang, Xue’e Zhang, Yuxi Wang, Yuhao Yang, Junchao Wei, Jian Yang

**Affiliations:** 1 School of Stomatology, Jiangxi Medical College, Nanchang University, Nanchang 330006, China; 17779145437@163.com (M.T.); 15949565767@163.com (M.G.); zhang04060217@163.com (X.Z.); 18802082298@163.com (X.Z.); wangyuxi0061@163.com (Y.W.); 15797835686@163.com (Y.Y.); 2 Jiangxi Provincial Key Laboratory of Oral Diseases, Nanchang 330006, China; 3 Jiangxi Provincial Clinical Research Center for Oral Diseases, Nanchang 330006, China; 4 School of Chemistry and Chemical Engineering, Nanchang University, Nanchang 330031, China

**Keywords:** dentin hypersensitivity, dentinal tubules, polydopamine, bioactive glass

## Abstract

When dentin is directly exposed to the oral cavity for various reasons, such as a lack of enamel on the tooth surface, external stimuli to the dentin often cause transient discomfort known as dentin hypersensitivity. In order to block the incoming stimulus signal, an ideal treatment is to induce the production of minerals to block the dentinal tubules. In this work, a dentin-desensitizing plugging material was prepared by modifying mesoporous bioactive glass with gelatin, the mineralization and desensitization effects of which were compared with Gluma in in vitro experiments. These experiments confirmed that gelatin-modified bioactive glass (MBG@PDA@Gel) is more effective than traditional desensitizing agents at blocking dentin tubules. Following the successful synthesis of MBG@PDA@Gel, as confirmed by scanning electron microscopy, transmission electron microscopy, and other tests, the treatment of demineralized dentin with MBG@PDA@Gel demonstrated that the dentinal tubules were tightly blocked under scanning electron microscopy. MBG@PDA@Gel induces minerals in deeper layers of dentinal tubules, promoting remineralization and forming a unified structure with the tubule blockage. Animal studies showed that MBG@PDA@Gel can remineralize demineralized dentin, and it is stable in the oral cavity and does not fall out. MBG@PDA@Gel not only enhances the biocompatibility of the nanoparticle but also results in an overall uniform and rapid remineralization of the demineralized dentin.

## 1. Introduction

Dentin hypersensitivity is a common disease with high clinical incidence, seriously impacting patients’ quality of life [1]. Dentin sensitization often occurs when dentin is directly exposed to the dynamic oral environment due to tooth brushing, the recession of the gums due to periodontal disease, and anatomical factors or medical factors that result in the loss of enamel on the surface of the teeth, or the loss of alveolar bone and periodontal tissue at the roots of the teeth [2,3]. This exposure can cause rapid, short-lived pain when exposed to external stimuli. The hydrodynamic theory, widely accepted by modern scientists, explains dentin hypersensitivity [4,5]. According to this theory, stimuli on the dentin surface are transmitted to the pulp tissue by multidirectional fluid flow within the dentinal tubules, exciting the pulpal nerve fibres and producing nociception.

Current treatments for dentin hypersensitivity fall into two categories: inhibition of the nerve conduction in and blocking of the dentinal tubules [6]. Clinical treatments that block nerve conduction, such as lasers and potassium ion desensitizers [7,8], provide only short-term relief and fail to achieve long-term therapeutic effects. The mechanism of laser desensitization involves the laser generating an instantaneous high heat field on the dentin surface, effectively melting inorganic and organic substances to block the dentinal tubules [9]. Nevertheless, the high heat field is often perceived as a potential source of damage and irritation to other tissues in the mouth. Accordingly, in selecting a safer desensitizer with long-term therapeutic benefits, dentinal tubule plugging materials are more closely aligned with this perspective.

While clinically used dentin desensitizers can seal dentinal tubules to some extent, they often fail to achieve long-term, stable sealing in the dynamic oral environment. For example, the Gluma desensitizer, which is commonly used in clinical practice, relies primarily on glutaraldehyde to induce the precipitation of plasma proteins in dentinal tubules, thereby protecting the pulp nerve from external stimuli [10,11]. Although the Gluma desensitizer was long considered effective for dentin hypersensitivity [12,13,14,15], Gluma desensitizers have poor resistance to acid etching and mechanical friction [16]. More importantly, Gluma desensitizers are not suitable for long-term desensitization in humans [17]. The ideal solution for dentin hypersensitivity involves creating a non-toxic, stable, and user-friendly material. More attention is being given to deep sealing materials that can induce mineralization to block dentinal tubules [18,19,20,21], while adhesive analogues designed to enhance the bond between the material and the remineralization interface are also available [22]. For example, using adhesive properties in treatments for demineralized dentin can ensure a tight bond between the material and the dentin interface, improving the remineralization layer’s resistance to mechanical wear and acid etching. This property proves the excellent prospect of bonding analogues with adhesive properties in the field of dentin allergy treatment and suggests a new direction for us to improve desensitizing nanoparticles.

Bioactive Glass (BG), used for bone regeneration in hard tissue restoration, has recently been used for dental tissue restoration [23,24,25]. Due to its biological activity, BG has demonstrated excellent therapeutic results in both pulp regeneration [26,27] and dental hard tissue remineralization [28,29]. The dentinal tubules are small tubules that taper from the pulpal end to the dentin surface, and previous studies showed that the average value of dentinal tubules in permanent teeth is 2.55–2.82 μm, which is an obstacle to the deep sealing of dentinal tubules [30]. Previous studies showed that BGs with a small particle size are more accessible to the dentinal tubules [31,32]. At the same time, size alterations offer additional benefits. It has been shown that changing the mesoporous structure and size of BGs improves binding properties and promotes the formation of mineralization products [33,34]. Consequently, the formation of mesoporous structure and pore volume increases the bioactivity and makes the nanoparticle more biologically active, which is demonstrated by the stronger hydroxyapatite-generating ability in mesoporous bioactive glass (MBG) nanoparticles compared to BGs [35]. Nevertheless, it remains to be determined whether MBG’s intrinsic capacity to impede mineralization can achieve the therapeutic effect of long-term dentinal tubule occlusion. According to the clinical requirements, it is necessary to increase the mineralization speed of MBG and enhance its adhesion to the dentin interface in order to develop an ideal dentinal tubule sealant.

**Figure 1:**
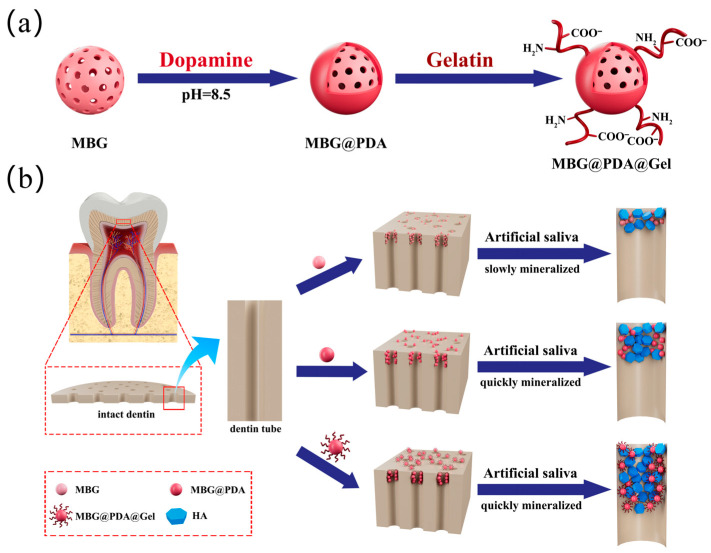
(**a**) Schematic diagram of MBG surface modification: (**b**) schematic illustration of the efficacy of three nanoparticles applied to occlude dentinal tubules.

In this work, novel nanoparticles were developed for the deep remineralization of dentinal tubules and the blocking of external stimulus signalling. The synthesis process of gelatin-modified MBG (MBG@PDA@Gel) is shown in Figure 1a. Firstly, a mussel-inspired approach was employed to treat MBG, resulting in the formation of Polydopamine(PDA)-coated MBG (MBG@PDA), which was then coated with gelatin to produce MBG@PDA@Gel. Due to the presence of gelatin molecules, MBG@PDA@Gel can penetrate deep into the dentinal tubules and induce mineralization quickly (Figure 1b). Both in vitro and in vivo results demonstrated that the minerals induced by MBG@PDA@Gel are much more stable when faced with acid erosion and mechanical friction, implying its long-term effect in treating dentin hypersensitivity.

## 2. Results and Discussion

### 2.1. Characterization of MBG and Surface-Modified MBG

**Figure 2:**
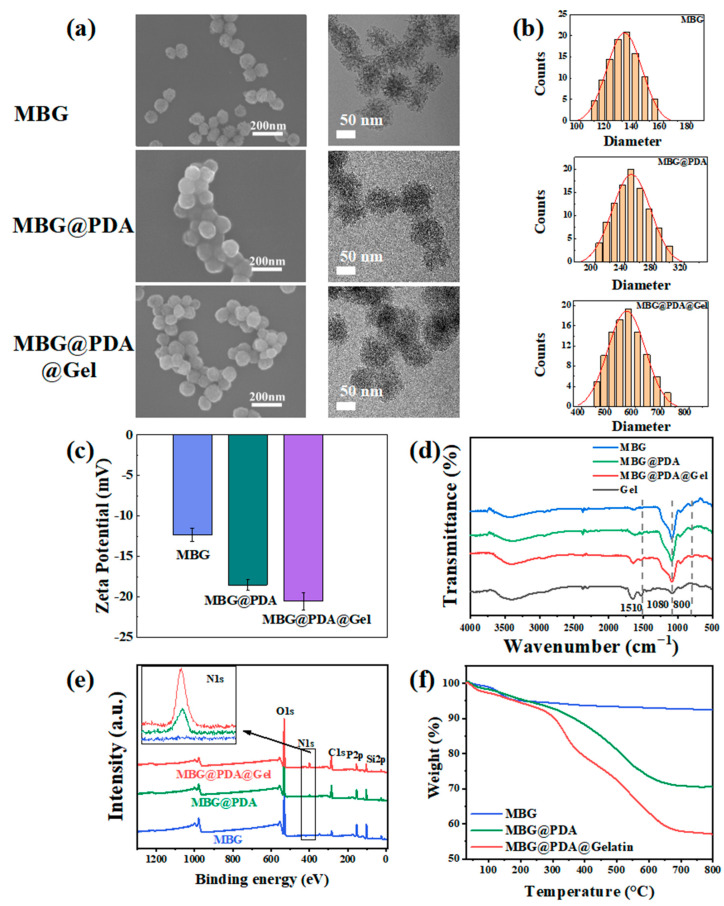
Material characterization: (**a**) Nanoparticle morphology. From left to right are SEM images, SEM local magnification, and TEM images. (**b**) Three nanoparticle particle size results. (**c**) Zeta potential diagrams of MBG, MBG@PDA, and MBG@PDA@Gel. (**d**) FTIR of MBG, MBG@PDA, MBG@PDA@Gel, and Gel. (**e**) XPS of MBG, MBG@ PDA, and MBG@PDA@Gel. (**f**) TGA of MBG, MBG@PDA, and MBG@PDA@Gel.

SEM images reveal that MBG, MBG@PDA, and MBG@PDA@Gel nanoparticles are spherical (Figure 2a). The surface of MBG nanoparticles is smooth, with a faintly discernible mesoporous structure, which is more clearly visible in TEM images, where it appears uniformly distributed within the spherical nanoparticles. Upon the adsorption of PDA onto the surface, a smooth layer forms, rendering the mesoporous structure of MBG unobservable in TEM. The hydrodynamic particle sizes of the nanoparticles increase with the adsorption of PDA and Gel, as measured by high-sensitivity zeta potential and a nanoparticle sizer (Figure 2b). Nevertheless, the dimensions of the MBG nanoparticles co-modified with PDA and gelatin remained considerably smaller than the diameter of dentinal tubules at this stage. Consequently, it was hypothesized that they could potentially penetrate the dentinal tubules. With the introduction of PDA and Gel, the negative potential of nanoparticles further decreases (Figure 2c). This decrease is due to the adsorption of PDA in an alkaline environment, where PDA exhibits a negative charge, thereby making the nanoparticles more negatively charged.

FTIR spectra reveal characteristic broad bands for MBG at about 800 cm⁻^1^ and 1080 cm⁻^1^, caused by Si-O and Si-O-Si vibrations, respectively (Figure 2d). The appearance of the N-H bond characteristic peaks occurred at about 1510 cm⁻^1^ in the spectra of MBG@PDA. And it was shown that the appearance of characteristic N-H peaks could confirm the successful adsorption of PDA on the MBG surface, as MBG itself does not contain amino groups [36]. The infrared spectrum of gelatin, showing characteristic peaks between about 1500–1750 cm⁻^1^, is also observed in the MBG@PDA@Gel spectra. The characteristic peaks of gelatin are mainly generated by the N-H stretching vibration of the amide bond and the coupling of the N-H bending vibration with the C-N stretching vibration [37]. XPS characterization further verified the successful synthesis of the two surface-modified nanoparticles (Figure 2e). The energy spectrum of MBG showed no N(1s) peak, whereas MBG@PDA exhibited an enhanced N(1s) peak due to the presence of amino groups in PDA. The N(1s) peak was further enhanced in MBG@PDA@Gel, confirming the successful modification of polydopamine and gelatin on the MBG surface.

In thermogravimetric analysis (TGA), weight loss in the temperature range of 30–180 °C is typically attributed to moisture in the sample (Figure 2f). The weight loss of nanoparticles under a nitrogen atmosphere in the range of 180–800 °C was analyzed, revealing a 3% weight loss for MBG. The polydopamine and gelatin adsorbed on the outer layer of MBG decompose during the temperature increase, resulting in weight losses of 26.6% for MBG@PDA and 40% for MBG@PDA@Gel. This change indicates the successful adsorption of substances on MBG at each step of the surface modification process.

### 2.2. Remineralization of Demineralized Dentin In Vitro

#### 2.2.1. Mineralized Morphology of Demineralized Dentin

**Figure 3:**
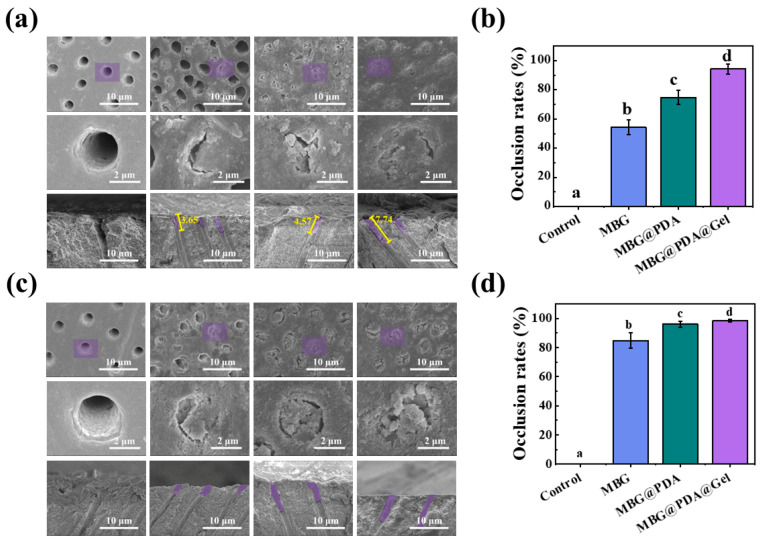
Dentinal tubule occlusion results of the Control, MBG, MBG@PDA, and MBG@PDA@Gel treatments after 1 day and 4 days. (**a**) SEM images of dentin surfaces and longitudinal sections at day 1 of mineralization. (**b**) Tubule blockage rate of dentin surfaces in each group at day 1 of mineralization. (**c**) SEM images of dentin surfaces and longitudinal sections at 4 days of mineralization. ((**a**,**c**) are Control, MBG, MBG@PDA, and MBG@PDA@Gel images from left to right, and in each group are the dentin disc surface image, the magnified image of the purple area of the surface image, and the longitudinal cross-section image of the dentin disc, from top to bottom, respectively). (**d**) Tubule blockage rate of dentin surfaces at 4 days of mineralization (with the same lowercase letters to indicate that the differences between each other are not statistically significant (*p* > 0.05)).

SEM images revealed that the dentinal tubules were open and structurally clear on the surface of acid-etched dentin discs (Figure S1). After 1 day of mineralization in artificial saliva (Figure 3a), the control group and the demineralized dentin treated with phosphoric acid displayed similar images with no clogging observed in the superficial dentinal tubules. In contrast, the MBG@PDA and MBG@PDA@Gel groups exhibited a significant clogging of dentinal tubules—in particular, the areas of dentinal tubules. The MBG group showed some blocked dentinal tubules, but many unobstructed dentinal tubules were still visible, with only a small portion blocked. By measuring the depth of dentinal tubule obstruction in longitudinal cross-sectional images, a noteworthy phenomenon was observed: the depths of obstruction in the MBG, MBG@PDA, and MBG@PDA@Gel groups were 3.65 μm, 4.57 μm, and 7.74 μm, respectively. This observation indicates that surface-modified MBG demonstrated an enhanced capacity to obstruct deep dentinal tubules after 1 day of mineralization. A computer analysis using Image J revealed that the dentinal tubule blockage rate (Figure 3b) after 1 day of mineralization was 51.5%, 70.1%, and 91.6% for the MBG, MBG@PDA, and MBG@PDA@Gel groups, respectively. During the same mineralization period, the introduction of polydopamine and gelatin significantly increased the number of atretic dentinal tubules, with a more uniform mineralized plugging of the surface of the demineralized dentin discs. Also, the overall plugging rate of dentinal tubules was enhanced. The outer gelatin layer in the MBG@PDA@Gel group enhanced nanoparticle penetration into dentinal tubules, potentially closing additional dentinal tubules, a phenomenon to be further explored in subsequent fluorescence experiments.

SEM images demonstrated greater dentinal tubule blockage on the fourth day of mineralization compared to the first day (Figure 3c). The MBG@PDA@Gel groups, which initially showed effective blockage, exhibited even further blockage at this time, with minimal unblocked areas. Figure 3d illustrates the dentinal tubule blocking rate at 4 days post-mineralization, showing an increase to 84.8% in the MBG group, while the MBG@PDA and MBG@PDA@Gel groups both exceeded 94%. This change indicates that, by the fourth day of mineralization, the three groups treated with MBG nanoparticles exhibited a higher level of dentinal tubule blocking.

**Figure 4:**
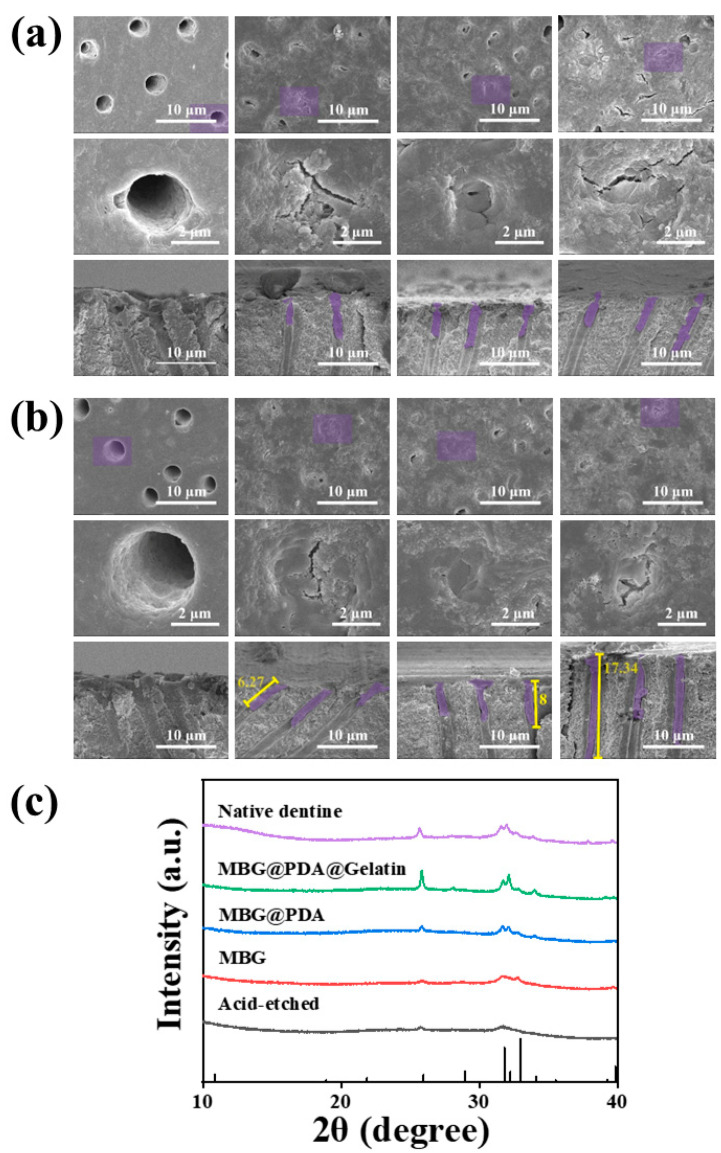
Dentinal tubule occlusion results for the Control, MBG, MBG@PDA, and MBG@PDA@Gel treatments after 7 days and 14 days. (**a**) SEM images of dentin surface and longitudinal section on day 7 of mineralization. (**b**) SEM images of the dentin surface and longitudinal section on day 14 of mineralization. ((**a**,**b**) are Control, MBG, MBG@PDA, and MBG@PDA@Gel images from left to right, and in each group are the dentin disc surface image, the magnified image of the purple area of the surface image, and the longitudinal cross-section image of the dentin disc, from top to bottom, respectively). (**c**) XRD images of the dentin surface for each group on day 14 of mineralization.

The mineralization time of dentin was extended to seven days. However, in the MBG, MBG@PDA, and MBG@PDA@Gel groups, some dentinal tubules remained partially unoccluded (Figure 4a). However, the degree of dentinal tubule closure at this time was significantly better than the desensitizer closure effect with fluoride-containing bioactive glasses and poly(catechols) [38,39]. Due to the accumulation of minerals on the surface, the dentinal tubules’ surfaces were obscured. Consequently, a follow-up work designed as a dentinal tubule penetration test was used to validate the overall mineralization and blockage effect. The longitudinal section of the dentinal tubules revealed tightly packed plugs. On day 14 of mineralization (Figure 4b), the MBG@PDA@Gel group showed the deepest blockage length compared to other groups, approximately 17.34 μm, which is twice the depth observed with MBG@PDA treatment.

X-ray diffraction (XRD) was used to confirm the formation of HA in each group of dentin discs mineralized for 14 days. On day 14, nascent mineral layers in the MBG group exhibited weak peaks at 2θ = 26°, 2θ = 31.8°, and 2θ = 32.2°, corresponding to the (002), (211), and (112) crystalline surfaces of HA, respectively. In the MBG@PDA group, these peaks were enhanced, and in the MBG@PDA@Gel group, the peaks were closer in intensity to normal dentin. This result confirmed the production of HA crystals in all three groups (Figure 4c). The calcium–phosphorus ratios of the dentin in each group after fourteen days of mineralization further supported these findings (Figure S2). The ratios for the demineralized, MBG, MBG@PDA, and MBG@PDA@Gel groups were 1.92, 1.88, 1.64, and 1.83, respectively, aligning closely with the HA calcium–phosphorus ratio.

#### 2.2.2. Immediate Distribution of Nanoparticles

**Figure 5:**
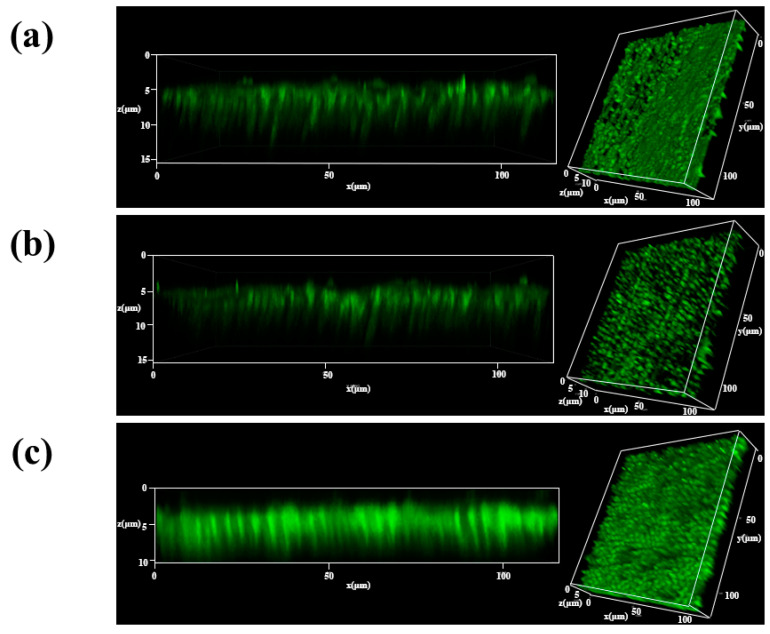
Laser confocal scanning of dentin surface and longitudinal section: (**a**) MBG group. (**b**) MBG@PDA group. (**c**) MBG@PDA@Gel group.

In order to immediately study the distribution of the three nanoparticles coated on the surface of demineralized dentin, we used fluorescence to label each of the three nanoparticles and then coated them on the surface of dentinal tubules according to the in vitro dentin disc mineralization treatment and observed them using laser confocal microscopy. The laser confocal analysis demonstrated no significant difference in the penetration depth of MBG, MBG@PDA, and MBG@PDA@Gel in dentinal tubules immediately after coating demineralized dentin. However, the MBG-treated dentin surface displayed non-uniform fluorescence, with some areas showing very low intensity, indicating sparse nanoparticle distribution (Figure 5a). In contrast, MBG@PDA exhibited a tendency to aggregate primarily within the dentinal tubule area, forming rounded dot-like fluorescence patterns. The longitudinal fluorescence analysis showed stronger fluorescence near the dentinal tubule mouths, indicating greater nanoparticle accumulation (Figure 5b). Enhanced fluorescence bands near the dentinal tubule surfaces suggested that nanoparticles adhered and aggregated in the superficial layers following PDA modification. The MBG@PDA@Gel group showed more uniform fluorescence on the dentin (Figure 5c). Additionally, the fluorescence intensity and density in the longitudinal section of the dentin disc were significantly higher than those in the MBG and MBG@PDA groups. This result suggests that more nanoparticles penetrated and occluded the dentinal tubules. Gelatin encapsulation increased the hydrophilicity of MBG@PDA, facilitating easier nanoparticle entry into the dentinal tubules. This action may be the reason why MBG@PDA@Gel can seal the dentinal tubules in a short time. The higher rate of dentinal tubule occlusion in the MBG@PDA@ Gel compared to the other groups at the same stage of mineralization can also be observed in the SEM images.

#### 2.2.3. Mechanical Properties

**Figure 6:**
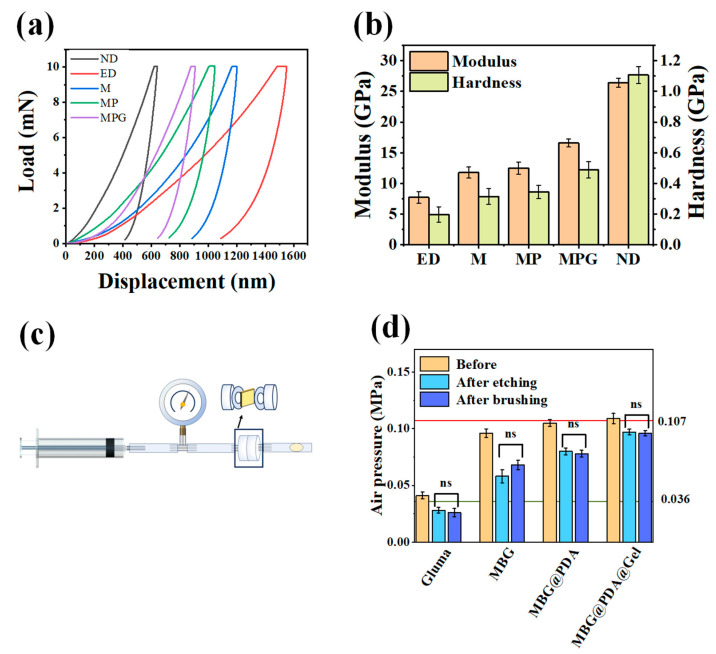
Test results of mechanical properties and dentin permeability: (**a**) Typical loading–unloading curves extracted from nanoindentation tests at remineralization time points. (ND: native dentin, ED: etched dentin, M: dentin treated by MBG, MP: dentin treated by MBG@PDA, MPG: dentin treated by MBG@PDA@Gel). (**b**) Elastic modulus and hardness of MBG, MBG@PDA, MBG@PDA@Gel, native dentin, and remineralized dentin matrix. (**c**) Schematic diagram of the air pressure test. (**d**) Air pressure graph of dentin slices treated with different desensitizer materials before and after brushing and subsequent etching (*n* = 3, ns = not significant. The red line indicates the air pressure values measured on natural dentin, while the green line indicates the air pressure values measured on acid-etched demineralized dentin).

The mechanical properties of the nascent mineral layer on the dentin surface after fourteen days of mineralization were tested using nanoindentation experiments. Under the same constant load, samples with poorer mechanical properties exhibited greater displacement. Among the four groups, the acid-etched group showed the deepest displacements, while the normal dentin group had the shallowest. The three experimental groups produced intermediate displacements, from the largest to smallest: the MBG group, followed by the MBG@PDA group, and the MBG@PDA@Gel group (Figure 6a).

Calculations of hardness and Young’s modulus indicated that the demineralized dentin’s mechanical properties were restored after nanoparticle treatment (Figure 6b). Following the acid etching treatment, the mechanical properties of dentin were found to have been adversely affected. The Young’s modulus was observed to have decreased from 26.41 GPa to 7.72 GPa, while the hardness decreased from 1.107 GPa to 0.198 GPa. Following nanoparticle treatment, Young’s modulus and hardness of the MBG group were 11.77 GPa and 0.315 GPa, Young’s modulus and hardness of the MBG@PDA group were 12.48 GPa and 0.344 GPa, Young’s modulus and hardness of the MBG@PDA@Gel group were 16.59 GPa and 0.488 GPa. Following the administration of the three nanoparticles, Young’s modulus and hardness of the dentin exhibited partial restoration. Specifically, the mineralized dentin surface in the MBG@PDA@Gel group exhibited mechanical properties closest to those of normal dentin. This outcome suggests that the mineralized layer in the MBG@PDA@Gel group achieved the highest degree of mineralization, closely approximating normal dentin.

#### 2.2.4. Occlusion of Dentinal Tubules

To further validate the sealing effect of MBG and two MBG surface-modified materials on dentinal tubules, a dentinal tubule penetration model was employed for each group of dentin discs (Figure 6c). The data indicated that, over a seven-day period, the pressure measured in the MBG, MBG@PDA, and MBG@PDA@Gel groups exhibited an increasing trend from left to right. Furthermore, the pressure values measured in the MBG@PDA@Gel group were found to be comparable to those observed in natural dentin. Consequently, it can be postulated that the MBG@PDA@Gel group produced a greater quantity of minerals, resulting in a dentin permeability that was most similar with that observed in natural dentin (Figure S3). The prolongation of the mineralization period to 14 days engenders a further augmentation in the air pressure values, which is accompanied by a reduction in dentin permeability. Nevertheless, the MBG@PDA@Gel group continues to exhibit the highest pressure values, which are even higher than those observed in natural dentin (Figure 6d). This result indicates reduced permeability and varying degrees of dentinal tubule blockage. Since the MBG, MBG@PDA, and MBG@PDA@Gel groups showed decreased permeability, it can be concluded that more minerals were generated in the MBG@PDA@Gel group, making its dentin permeability closest to that of natural dentin. Extending the mineralization time to 14 days further increased the measured air pressure values, resulting in even lower dentin permeability, with values approaching those of normal dentin.

#### 2.2.5. Dentinal Tubule Sealing Stability

Complex changes in the oral environment, such as mechanical friction and pH fluctuations due to chewing, eating, and daily cleaning, present significant challenges to the treatment of dentin hypersensitivity. To achieve long-lasting and stable desensitization, novel agents must be developed to withstand these challenges without degradation. To simulate the dynamic oral environment, two protocols were employed: acid erosion with citric acid [40] and mechanical friction using a soft-bristled toothbrush. These tests assessed the dentinal tubule sealing efficacy of each nanoparticle group in tolerating the complex conditions of the oral cavity.

**Figure 7:**
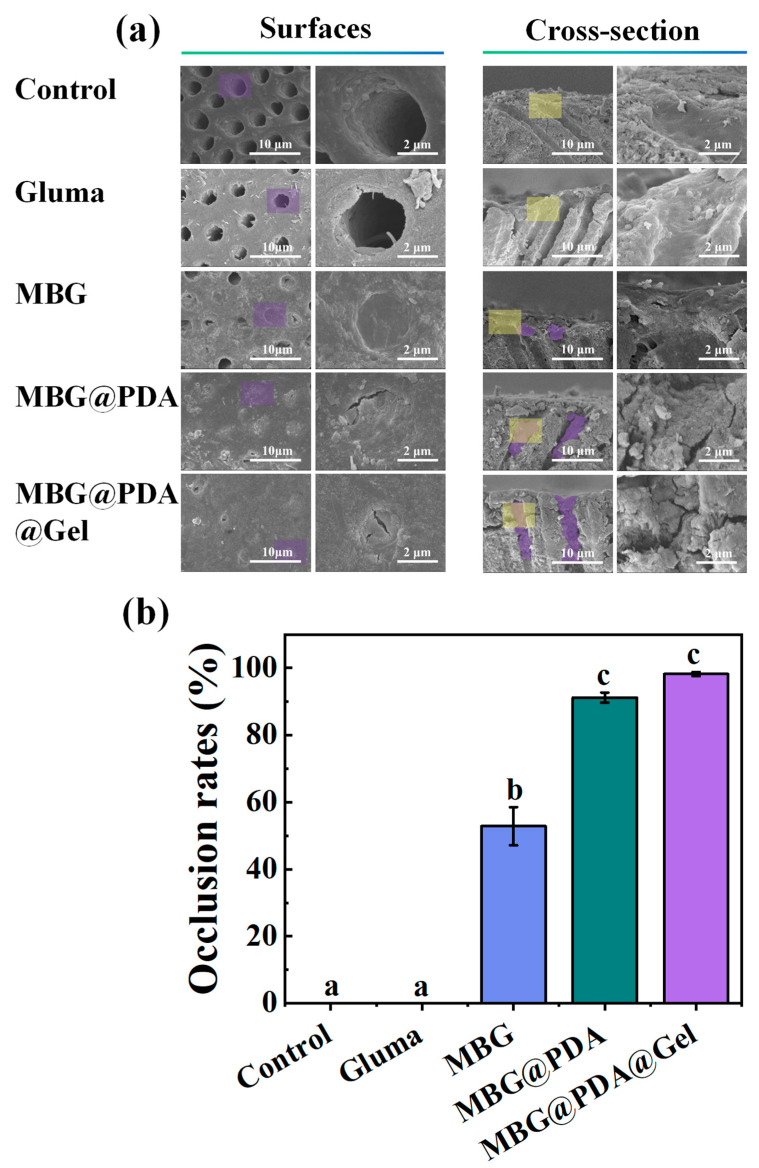
Closure of dentinal tubules after acid etching treatment of dentinal tubules in each group. (**a**) SEM images of the lower surface and cross-section of the dentinal tubules in the control group and the Gluma, MBG, MBG@PDA, and MBG@PDA@Gel treatment after acid etching (In the SEM image of the surface and cross-section images, the right image is the zoomed-in image corresponding to the purple and yellow areas in the left image, respectively). (**b**) The dentinal tubule clogging rate of each group of dentin after acid erosion (Using the same lowercase letters indicates that the differences between each other are not statistically significant (*p* > 0.05)).

SEM revealed that demineralized dentin discs treated with a Gluma desensitizer formed a relatively loose sediment layer on the dentin surface, partially blocking some dentinal tubules (Figure S4). However, dentin permeability measurements indicated only a slight alteration in permeability after treatment with Gluma. After citric acid erosion, the originally blocked dentinal tubules reopened, suggesting that this blockage is fragile and short-lived in the actual oral environment (Figure 7a). In the Gluma group, dentin permeability increased after acid erosion, surpassing that of the control group (Figure 6d). This phenomenon likely occurs because the acid etching reopens the previously blocked dentinal tubules and further demineralizes the unblocked ones. In the MBG group, acid etching dissolved the mineral rims of the originally blocked dentinal tubules, leading to the partial or complete reopening of most dentinal tubules. Conversely, in the MBG@PDA@Gel groups, despite acid etching, the dentinal tubules remained tightly sealed, with citric acid causing only minor damage to the superficial neo-mineralized dentin layer. Calculating the sealing rate after acid etching revealed that, unlike the conventional Gluma desensitizer, the sealing rate remained high in the MBG@PDA and MBG@PDA@Gel groups. Acid etching did not significantly affect the neoplastic minerals blocking the dentinal tubules. Dentin permeability measurements showed the smallest change in permeability in the MBG@PDA@Gel group, followed by the MBG@PDA group (Figure 7b). This observation confirms that MBG surface modification effectively enhances resistance to acid erosion, improving the treatment of dentin hypersensitivity. The MBG@PDA@Gel group exhibited greater acid resistance due to deeper dentinal tubule occlusion than other groups by the produced minerals.

**Figure 8:**
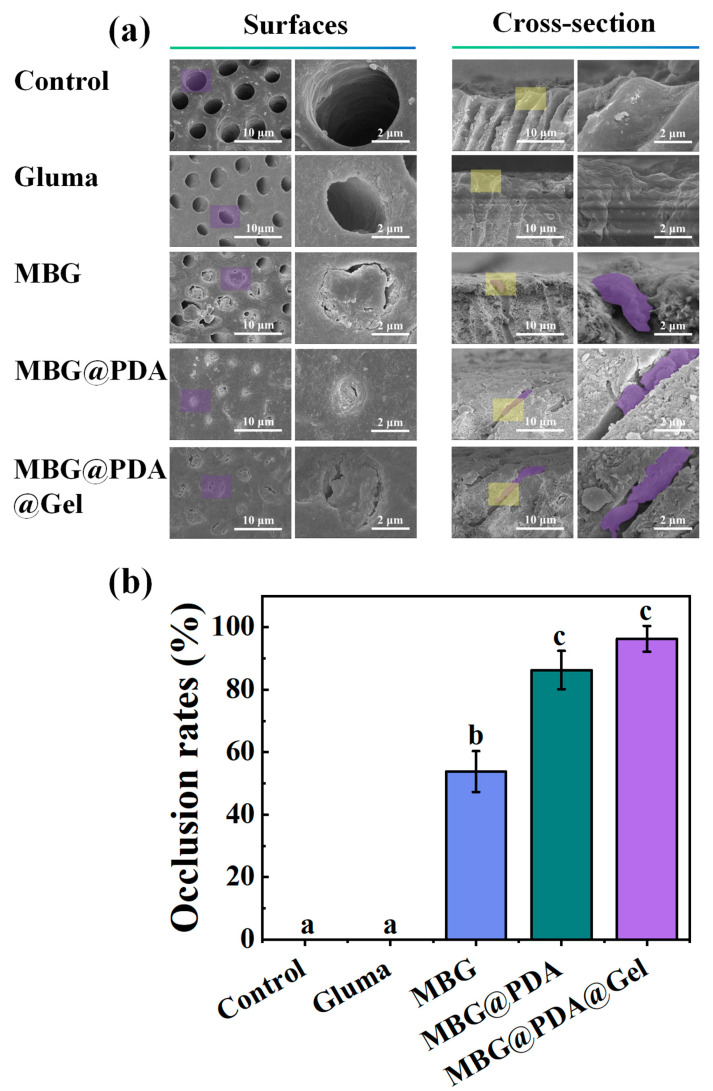
Closure of dentinal tubules after mechanical friction of dentinal tubules in each group. (**a**) SEM images of the surface and cross-section of the dentinal tubules after mechanical friction in the control group and after treatment with Gluma, MBG, MBG@PDA, and MBG@PDA@Gel (In the SEM image of the surface and cross-section images, the right image is the zoomed-in image corresponding to the purple and yellow areas in the left image, respectively). (**b**) Dentinal tubule clogging rate of each group of dentin after mechanical friction (With the same lowercase letter to indicate that the difference between each other is not statistically significant (*p* > 0.05)).

After mechanical treatment with a soft-bristled toothbrush, the collagen fibres of control demineralized dentinal tubules partially collapsed, leaving the dentinal tubules open (Figure 8a). The Gluma-treated dentin did not exhibit clogging on the surface or in the cross-section, confirming that the simple protein denaturation of clogged dentinal tubules cannot withstand the mechanical friction of chewing or daily cleaning over time. In contrast, the MBG@PDA and MBG@PDA@Gel groups maintained tight blockage in the lumen despite some dentinal tubule edge damage from friction. However, dentin permeability measurements showed that the MBG@PDA@Gel group had lower permeability than the MBG@PDA group after mechanical friction treatment (Figure 8b), indicating superior clogging stability under mechanical stress.

From the acid etching and mechanical friction experiments, it is evident that, while gelatin surface modification did not significantly increase the depth of nanoparticle penetration into dentinal tubules, the mineral layer produced by MBG@PDA@Gel demonstrated better resistance to acid etching and mechanical friction compared to MBG nanoparticles alone. SEM showed that most the dentinal tubules of MBG@PDA@Gel-treated dentin discs after acid etching or mechanical friction remained occluded and were more resistant to acid and mechanical friction treatment compared to previous studies [16,41]. The enhanced sealing and resistance to acid erosion and mechanical friction observed in the MBG@PDA@Gel group can be attributed to the tight plugging of the deeper parts of the dentinal tubules by the minerals produced.

### 2.3. Remineralization of Demineralized Dentin In Vivo

**Figure 9:**
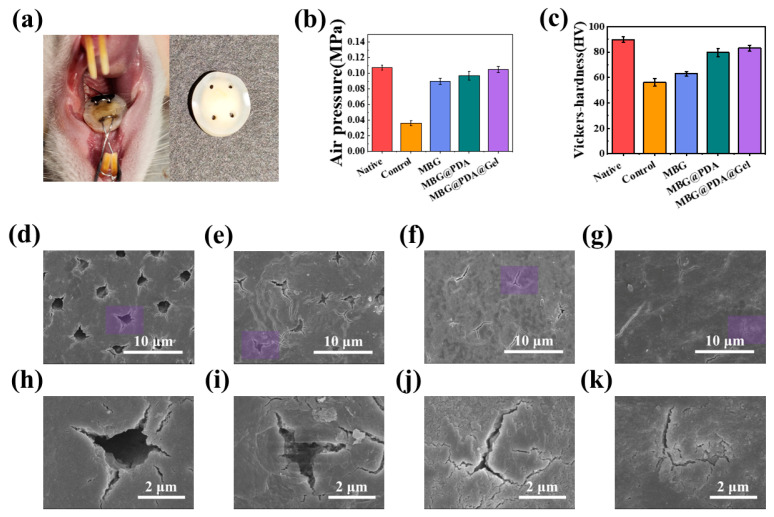
Remineralization of demineralized dentin in vivo. (**a**) Dentin disc is fixed in the oral cavity of a rat. The dentin disc is fixed with ligature wire between the maxillary anterior teeth and molars in rats’ oral cavities. (**b**) Results of air pressure tests to measure the permeability of different dentin samples in vivo. (**c**) Results of Vickers hardness of different dentin samples in vivo. (**d**–**g**) SEM images of the dentin surface in each group after 14 days of in vivo mineralization. (**d**): Control, (**e**): MBG, (**f**): MBG@PDA, (**g**): MBG@PDA@Gel. (**h**–**k**) are magnified images of the purple regions in (**d**–**g**).

To verify the effects of the three treatments in an in vivo environment, experiments were conducted using Sprague Dawley male rats. Untreated demineralized dentin discs and those treated with MBG and two types of surface-modified MBG were placed in the oral environment of rats to observe dentinal tubule occlusion after 14 days of mineralization (Figure 9a). The rat oral cavity, which contains various enzymes and ions, closely simulates the human oral environment. An SEM analysis showed that all dentinal tubules in the control group remained open after 14 days. Although MBG-treated dentin produced minerals, high-magnification SEM revealed that the open fissures of dentinal tubules were not completely closed (Figure 9i). In contrast, the MBG@PDA and MBG@PDA@Gel groups exhibited blocked dentinal tubules on the dentin surface, with a thicker mineral layer forming, obscuring the original dentinal tubule contours (Figure 9f,g,j,k).

However, measurements using a dentinal tubule permeability device showed a significant decrease in dentin permeability in all three treatment groups after mineralization, with MBG@PDA@Gel still the most effective at blocking dentinal tubules, in line with the in vitro results (Figure 9b). This result suggests that MBG@PDA@Gel effectively occludes dentinal tubules under in vivo conditions. It was also demonstrated that drugs can still function in the oral environment to tightly plug dentinal tubules, independent of activities such as chewing. After 14 days of mineralization, the permeability of demineralized dentin treated with MBG@PDA@Gel was similar to that of natural dentin, highlighting the therapeutic effectiveness of these nanoparticles. Vickers hardness testing of the newly mineralized layer showed that, although the MBG group had increased hardness compared to untreated demineralized dentin, it did not approach the hardness of natural dentin (Figure 9c). However, the mineralized layer formed after treatment with MBG@PDA@Gel had hardness values very similar to natural dentin. This observation indicates that the mechanical properties of demineralized dentin were restored to near-healthy levels with these treatments. Thus, the introduction of gelatin significantly enhanced the ability of MBG nanoparticles to occlude dentinal tubules.

### 2.4. Biocompatibility

As a dentin desensitizer for intraoral use, it is not sufficient for the desensitizing effect to be merely satisfactory. In this study, we sought to ascertain the cytotoxicity and organotoxicity of the nanoparticle in order to verify its good biocompatibility.

#### 2.4.1. Cytotoxicity

**Figure 10:**
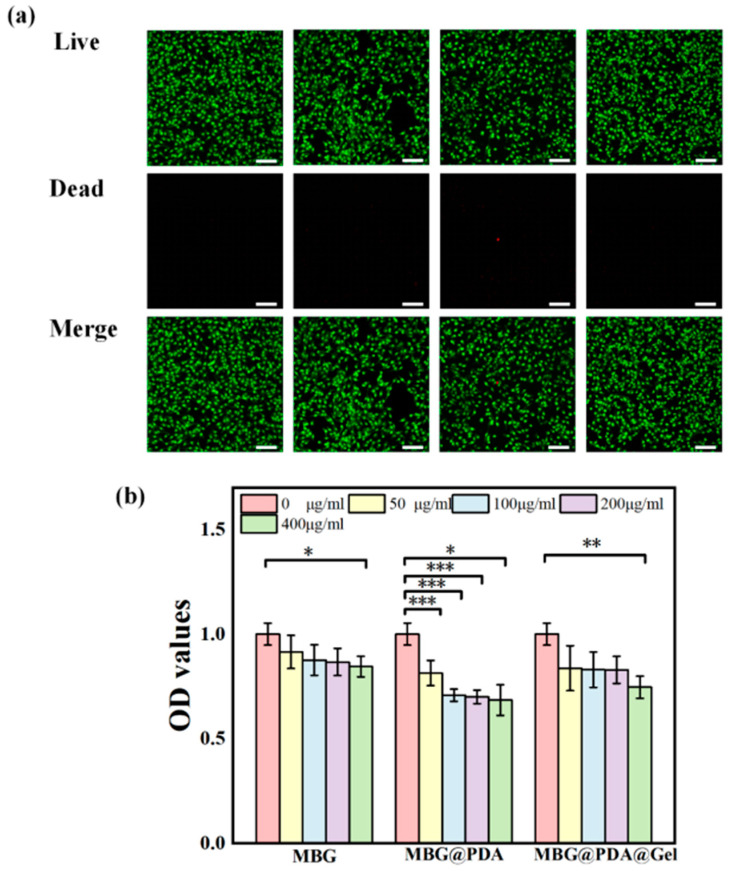
(**a**) MC3T3-E1 cell viability staining. Scale bar = 100 μm. (**b**) CCK-8 results of MC3T3-E1 cell. * *p* < 0.05, ** *p* < 0.01, *** *p* < 0.001.

**Figure 11:**
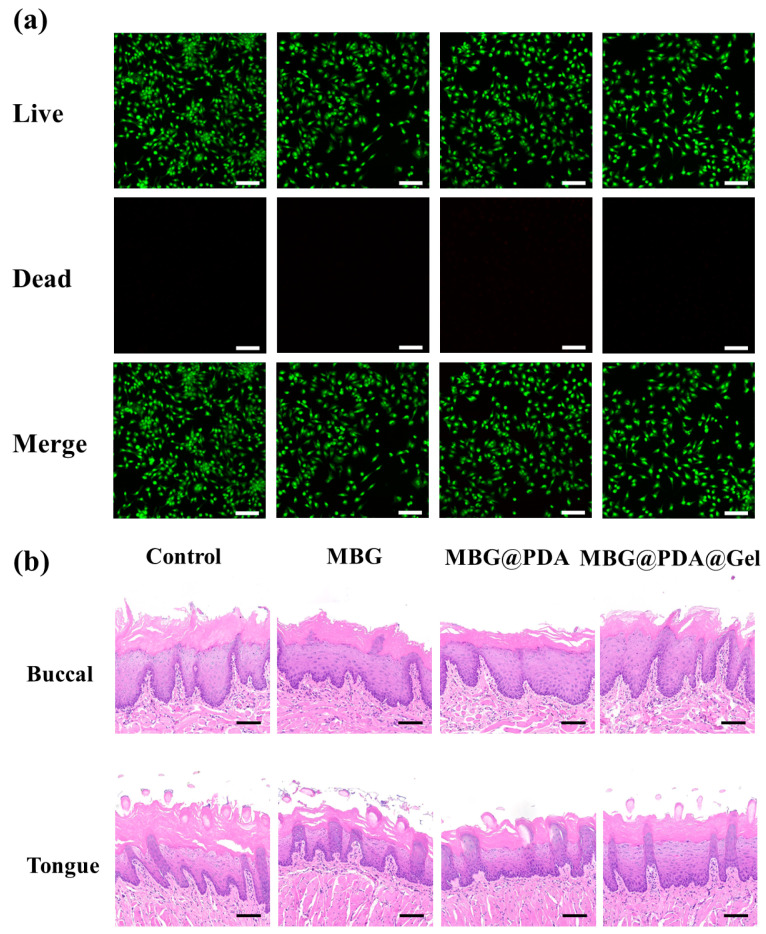
(**a**) L929 cell viability staining. Scale bar = 100 μm. (**b**) HE staining of buccal and lingual mucosa in rats. Scale bar = 100 μm.

MC3T3-E1 was used in this study, co-cultured with concentrations of 50, 100, 200, and 400 μg/mL of three kinds of nanoparticles for 24 h and then stained to observe cell survival (Figure S5 and Figure 10a). Live–dead staining results showed that, even at a nanoparticle concentration of 400 μg/mL, virtually no dead MC3T3-E1 cells were observed, confirming that MBG and its surface-modified MBG nanoparticles do not significantly harm these cells, allowing them to survive (Figure 10a). Considering that the clinical application of nanoparticles on the dentin surface will inevitably contact oral mucosal tissues, L929 cells were also used for co-culture and staining, yielding similar results to MC3T3-E1 cell staining, with all groups showing good biocompatibility (Figure 11a and Figure S6).

Additionally, the cytotoxic effects of MBG and two surface-modified nanomaterials on MC3T3-E1 were investigated using the CCK-8 assay (Figure 10b). MC3T3-E1 cells were incubated in a complete culture medium containing different doses of MBG nanoparticles for 24 h. A very slight decrease in cell activity was detected only at a concentration of 400 μg/mL, with the relative cell survival rate still above 85%. The introduction of polydopamine resulted in a decreasing trend in cell viability for MBG@PDA, yet 69% relative cell viability was maintained even at high concentrations. Coating MBG@PDA with a gelatin shell significantly improved cell survival compared to MBG@PDA, with relative cell survival at 400 μg/mL increasing to 75%. This improvement is attributed to the good biocompatibility of gelatin, which mitigated the microtoxicity of the nanoparticles.

#### 2.4.2. Tissue and Organ Toxicity

As a dentin-desensitizing material for intraoral use, it is crucial that it does not exert toxic effects on tissue organs. To assess organ toxicity, this study conducted further evaluations. After 14 days of mineralization with intraorally fixed dentin discs, the buccolingual tissues in contact with the discs were collected for HE tissue staining to verify non-toxicity by analyzing inflammation within the tissues. Figure 11b shows that the buccolingual tissues of rats treated with nanoparticles were similar to those of the control group. The tissues in all groups had an intact complex squamous epithelium, and the epithelial structure remained clearly visible. No inflammatory cell infiltration or other lesions were observed in the epithelium or lamina propria, confirming that the nanoparticles were non-toxic to the mucosal tissues. Furthermore, this study investigated whether the nanoparticles caused any damage or harm to the rats’ organs, given that the nanoparticle-treated dentin discs were placed in the rats’ oral cavities for 14 days. Hearts, livers, and kidneys from the rats treated with MBG and the two surface-modified MBG nanoparticles were extracted for HE tissue staining. The staining showed no inflammation or other pathological phenomena in the organs of any group (Figure S7), confirming the good biocompatibility and non-toxicity of MBG and the two surface-modified materials.

## 3. Materials and Methods

### 3.1. Reagents

Chloramine T, cetyltrimethylammonium bromide (CTAB), 3-aminopropyltriethoxysilane (APTES), dopamine hydrochloride (DA), citric acid, and N-2-hydroxyethylpiperazine-N′-2-ethanesulfonic acid (HEPES) were obtained from Sigma-Aldrich. Tetraethyl orthosilicate (TEOS), ethyl acetate (EA), ethanol, and anhydrous calcium chloride were purchased from Sinopharm. The chemicals ammonia, triethyl phosphate (TEP), and calcium nitrate tetrahydrate (CN) were procured from Macklin.

### 3.2. Synthesis of Bioactive Glass

MBG nanoparticles were synthesized following a previous method [42]. First, 0.7 g of CTAB was dissolved in 33 mL of deionized water using ultrasonic dispersion. The solution was then stirred magnetically while 10 mL of ethyl acetate was added and stirred for 30 min to form microemulsion droplets. Next, 7 mL of 1 mol/L ammonia water was added and stirred for 15 min. Subsequently, 3.6 mL of TEOS, 0.36 mL of TEP, and 2.277 g of calcium nitrate tetrahydrate were added sequentially every 30 min. After vigorous stirring for 4 h, the resulting white precipitate was collected by centrifugation, washed three times alternately with ethanol and ultrapure water, and freeze-dried to obtain a white powder. Finally, the powder was calcined in a muffle furnace at 650 °C for 6 h to remove the template and obtain MBG.

### 3.3. Synthesis of Surface-Modified MBG

First, 200 mg of MBG nanoparticles were ultrasonically dispersed in 100 mL of Tris-HCl (0.05 M, pH 8.5) for 30 min [43]. After complete dispersion, 200 mg of dopamine hydrochloride was added. The mixture was stirred continuously with a magnetic stirrer at room temperature (400–500 RPM). After 12 h, the mixture was centrifuged at 9000 RPM for 5 min to collect the sediment. The sediment was then washed three times with deionized water and lyophilized to obtain MBG@PDA nanoparticles.

A total of 200 mg of gelatin was dissolved in 50 mL of deionized water at 50 °C and then mixed with 50 mL of MBG@PDA solution. After 12 h of stirring, the product (MBG@PDA@Gel) was collected via centrifugation and then washed three times with deionized water and lyophilized.

### 3.4. Fluorescence-Labelled Material

Initially, MBG was amino-functionalized using APTES. A quantity of 0.3 g of MBG nanoparticles was dissolved in 60 mL of absolute ethanol and subjected to ultrasonic dispersion. Subsequently, 0.6 mL of APTES was added, followed by refluxing at 80 °C for 24 h. The sediment was collected, washed three times with deionized water, and freeze-dried to yield MBG@NH_2_.

Next, 30 mg of MBG@NH_2_ was dissolved in 10 mL of anhydrous ethanol and evenly dispersed. Then, 1.5 mg of FITC was added and stirred with a magnetic stirrer for 24 h in the dark. After centrifugation at 9000 RPM for 5 min, the solution was washed three times and then dried using a freeze dryer. MBG@PDA and MBG@PDA@Gel were fluorescently labelled using the same method as MBG@NH_2_.

### 3.5. Preparation of Demineralized Dentin Discs

Upper and lower third molars extracted from the Affiliated Stomatological Hospital of Nanchang University were collected. The ethics committee of Nanchang University’s affiliated stomatological hospital reviewed and approved both the collection of human teeth and the experimental plan. The teeth were placed in a 0.5% chloramine T solution and sonicated for 15 min after the removal of surface calculus and soft tissue. At the interface of enamel and cementum, the crown was separated from the tooth using a low-speed, water-cooled diamond saw to cut out the dentin disc, obtaining a 1 mm thick sample of the dentin disc at the pulp chamber. The samples were sequentially polished with SiC papers (600, 1000, 1500, and 2000 grain sizes) under running water. The dentin was etched with 35% phosphoric acid gelatin for 30 s, rinsed with deionized water, and sonicated for 15 min. The treated dentin discs were stored in deionized water at 4 °C for future use.

### 3.6. Characterization

The surface morphology and ultrastructure of the three materials were examined using transmission electron microscopy (TEM) at 5 kV and scanning electron microscopy (SEM). The particle size and zeta potential of the materials were determined using a high-sensitivity zeta potential and particle size analyzer. Nanoparticle structure and functional groups were analyzed using Fourier transform infrared spectroscopy (FTIR). The mass loss of the materials from 30 °C to 800 °C was measured at a rate of 10 °C/min under a nitrogen atmosphere using a thermogravimetric analyzer (TGA). The surface elemental composition was analyzed using X-ray photoelectron spectroscopy (XPS).

### 3.7. In Vitro Remineralization

#### 3.7.1. Mineralized Morphology of Demineralized Dentin

MBG, MBG@PDA, and MBG@PDA@Gel were applied to the demineralized dentin discs at a concentration of 20 mg/mL, and the dentin discs were coated using deionized water as a control group. After rinsing with deionized water, the treated dentin sections of each group were immersed in artificial saliva maintained at 37 °C and replaced with fresh artificial saliva every 24 h. SEM images of the cross-section and surface of the dentin discs were acquired from each group on days 1, 4, 7, and 14 of mineralization.

The calcium–phosphorus ratio data for each group were subjected to analysis via EDS. The XRD analysis on day 14 samples confirmed whether the crystal material blocking the dentinal tubules was hydroxyapatite. Three SEM images of dentin surfaces from each group of dentin after 14 days of mineralization were analyzed for the dentinal tubule sealing rate. The areas of dentinal tubules and the occluded areas were measured and statistically analyzed with Image J software version 1.54g. The occlusion rate for each image was calculated, and the average of the three sets of data provided the occlusion rate for each group.

#### 3.7.2. Immediate Distribution of Nanoparticles

Fluorescently labelled nanoparticles were applied to the surface of demineralized dentin discs at a concentration of 20 mg/mL, with each application lasting 1 min and repeated three times (*n* = 3). After drying, the dentin discs were rinsed with deionized water and then dried in a vacuum drying oven. The treated dentin discs were subsequently imaged using a laser confocal scanning electron microscope.

#### 3.7.3. Mechanical Properties

Each dentin disc remineralized for 14 days, along with healthy dentin discs, were tested with nanoindentation (Anton Paar UNHT, Graz, Austrian) after drying. The loading duration was 10 s, with a holding time of 2 s, a Poisson’s ratio of 0.28, and an applied force of 10 mN. Each sample was tested at ten points, with three samples in each group. The resulting data were analyzed to generate load–displacement curves, and the elastic modulus and hardness of each group were calculated.

#### 3.7.4. Occlusion of Dentinal Tubules

For each dentin disc after 14 days of remineralization, along with healthy dentin discs without acid etching, air pressure was measured (*n* = 3) using the dentinal tubule permeability model. Each sample underwent five air tightness tests. The data were analyzed and processed to create a histogram of the dentin disc permeability. During the test, the dentin disc was placed between two “O”-shaped silicone films, and the pressure value was read from the pressure gauge when the droplets in the hose on the right side of the dentin disc moved [44]. The measured data were tested using one-way ANOVA, followed by Tukey’s post hoc test.

#### 3.7.5. Dentinal Tubule Sealing Stability

The acid corrosion resistance and mechanical friction resistance experiments were designed to simulate the internal conditions of the human mouth. They were used to measure the effectiveness of nanoparticles in sealing dentinal tubules under acid corrosion or mechanical friction.

Acid Resistance: After 14 days of remineralization, four groups of samples underwent acid erosion treatment with a 6% citric acid solution for 60 s (*n* = 3). The occlusion of dentinal tubules was compared under SEM before and after the treatment. Additionally, the dentinal tubule permeability of the four groups post-acid etching was measured using the method described in Section 3.7.4.

Mechanical Friction Resistance: Four groups of samples, following 14 days of remineralization, were subjected to mechanical brushing with a soft toothbrush for 3 min (*n* = 3). The occlusion of dentinal tubules was compared under SEM before and after brushing. Furthermore, the dentinal tubule permeability of the four groups post-mechanical brushing was measured using the method outlined in Section 3.7.4.

### 3.8. In Vivo Mineralization

Human dentin specimens were fabricated into 1 mm thick discs and secured within the oral cavities of 8-week-old male Dawley rats (220–300 g, *n* = 5 per group). Four holes were drilled into each disc for fixation, and the edges were smoothed and rounded to prevent oral abrasions. The dentin discs were etched with 35% phosphoric acid to create artificial demineralized dentin. Treatment of the discs followed the protocol used in the in vitro mineralization section, with control samples treated with deionized water. Rats were anesthetized using intraperitoneal injections of 1% barbital sodium (0.4 mL/100 g). A 0.25 mm stainless steel ligature was inserted between the first and second molars, attached to the dentin disc, and connected to the upper central incisor. To mimic dentinal tubule occlusion in the human oral cavity, the dentin surfaces were exposed to natural rat saliva, containing various enzymes and ions. The rats’ diet was powdered to prevent mechanical damage to the specimens.

After 14 days of mineralization, the dentin discs were harvested, rinsed with deionized water, and examined using SEM. Air pressure values for the dentin discs were measured using a dentin permeability model. The hardness of the samples was assessed with a Vickers hardness tester, and the results were represented in a histogram.

### 3.9. Biocompatibility

#### 3.9.1. Cytotoxicity

All the nanoparticles were disinfected by immersion in 75% ethanol, and, after complete evaporation of the ethanol, the nanoparticles were ultrasonically dispersed in a DMEM high-glucose medium, and solutions of the following concentrations were prepared for subsequent cell co-culturing: 0 μg/mL, 50 μg/mL, 100 μg/mL, 200 μg/mL, and 400 μg/mL. The 3T3-E1 rat embryo osteoblast (MC3T3-E1) cells were seeded at a density of 2 × 10^4^ cells/mL into 96-well plates. After overnight incubation, the cells were co-cultured with the materials for 24 h, with six parallel wells set for each concentration. Following 24 h of co-culturing, fresh CCK-8 was added to each well, and the OD value was measured at 450 nm after 2–4 h of dark incubation. Simultaneously, cell viability staining was performed on cells co-cultured with the materials for 24 h at various concentrations, observing the ratio of viable to dead cells to assess material toxicity. Both mouse fibroblast cells (L929 cells) and MC3T3-E1 cells underwent cell viability staining in this study.

#### 3.9.2. Tissue and Organ Toxicity

In animal experiments, rats were sacrificed after 14 days of mineralization, with demineralized dentin discs fixed in their mouths. Some discs were treated with three types of nanoparticles, while others remained untreated. The buccal and lingual mucosa, heart, liver, and kidneys of the rats, which were in direct contact with the dentin discs, were observed and sampled for HE staining analysis. This analysis aimed to determine whether the nanoparticles caused toxic effects on the rats’ tissues and organs during the in vivo mineralization process.

## 4. Conclusions

In conclusion, MBG@PDA@Gel, a dentin desensitizer with excellent biocompatibility, was prepared through a surface modification of MBG using gelatin. MBG@PDA@Gel uniformly occludes deep dentinal tubules, and these low-toxicity nanoparticles can be rapidly mineralized, forming a mineralized layer that can restore the mechanical properties of demineralized dentin close to that of native dentin. The plug formed by MBG@PDA@Gel is more resistant to acid etching and mechanical friction than that of conventional Gluma desensitizers, achieving long-term stable dentinal tubule occlusion. Meanwhile, in vivo studies have demonstrated the safe, stable, and long-lasting therapeutic effect of MBG@PDA@Gel in the oral environment, proving their potential for clinical treatment.

## Data Availability

Data are contained within this article and Supplementary Materials.

## Supplementary Materials

The supporting information can be downloaded at: https://www.mdpi.com/article/10.3390/ijms252211867/s1.
