# Supplementary material for: Gelatin-Modified Bioactive Glass for Treatment of Dentin Hypersensitivity
Source: Int J Mol Sci. 2024 Nov 5;25(22):11867. doi: 10.3390/ijms252211867 (PMC11593888; doi:10.3390/ijms252211867)
Supplement: Supplementary file 1 [file ijms-25-11867-s001.zip › ijms-3289038-supplementary.pdf]

# Supporting Information

# Gelatin-modified bioactive glass for treatment of dentin hypersensitivity

*Mengzhen Tang, Min Ge, Xu Zhang, Xuee Zhang, Yuxi Wang, Yuhao Yang, Junchao Wei*, Jian Yang**

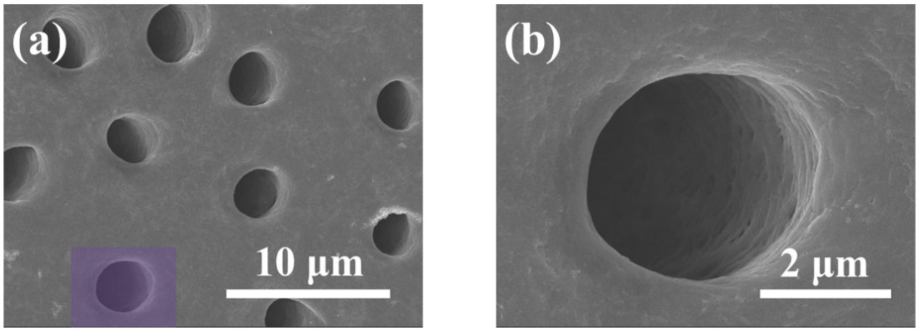


**Figure S1.**The SEM of Demineralized dentin. (b) An enlarged image of the purple area in (a)

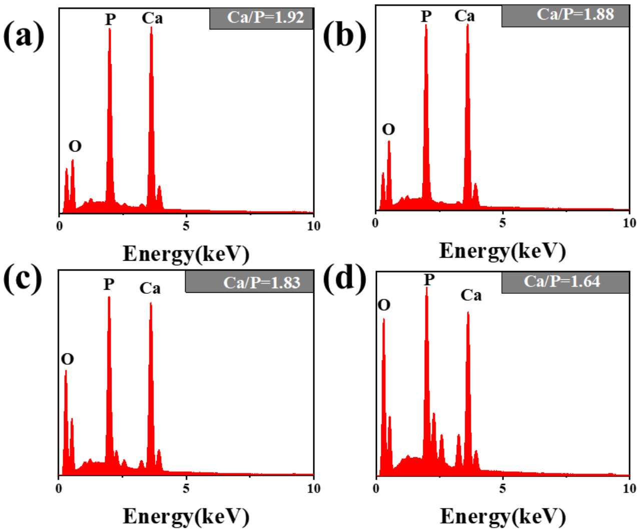


**Figure S2.** Calcium phosphorus ratio of newly formed mineral layers in the dentin of each group after 14 days of mineralization (a) Control group (b) MBG group (c) MBG@PDA Group (d) MBG@PDA @Gel group

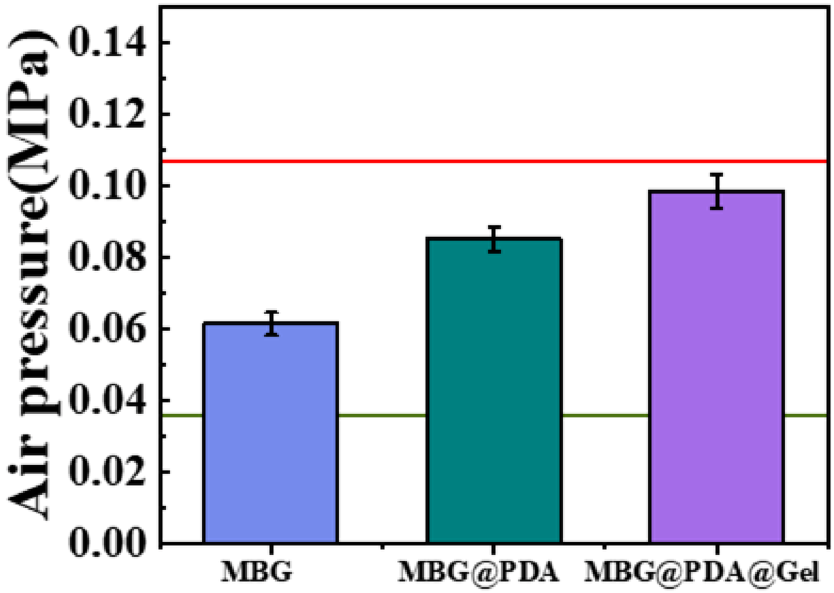


**Figure S3.** Air pressure values after 7 days of mineralization of dentin discs after the last three nanoparticles treatments. (The red line indicates the air pressure values measured on natural dentin, while the green line indicates the air pressure values measured on acid-etched demineralized dentin.)

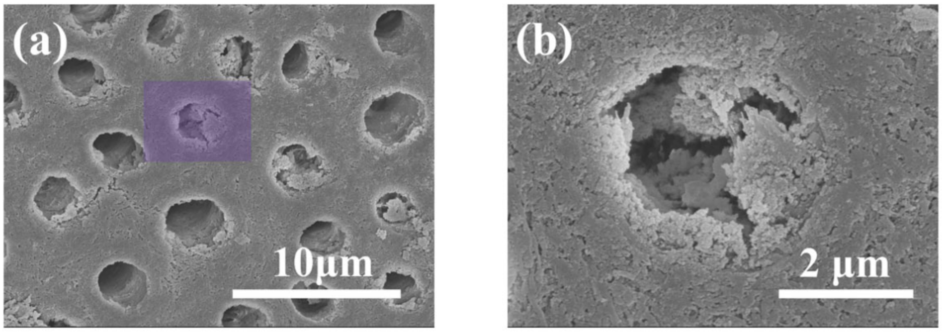


**Figure S4.** The SEM of dentin after Gluma treatment. (b) An enlarged image of the purple area in (a)

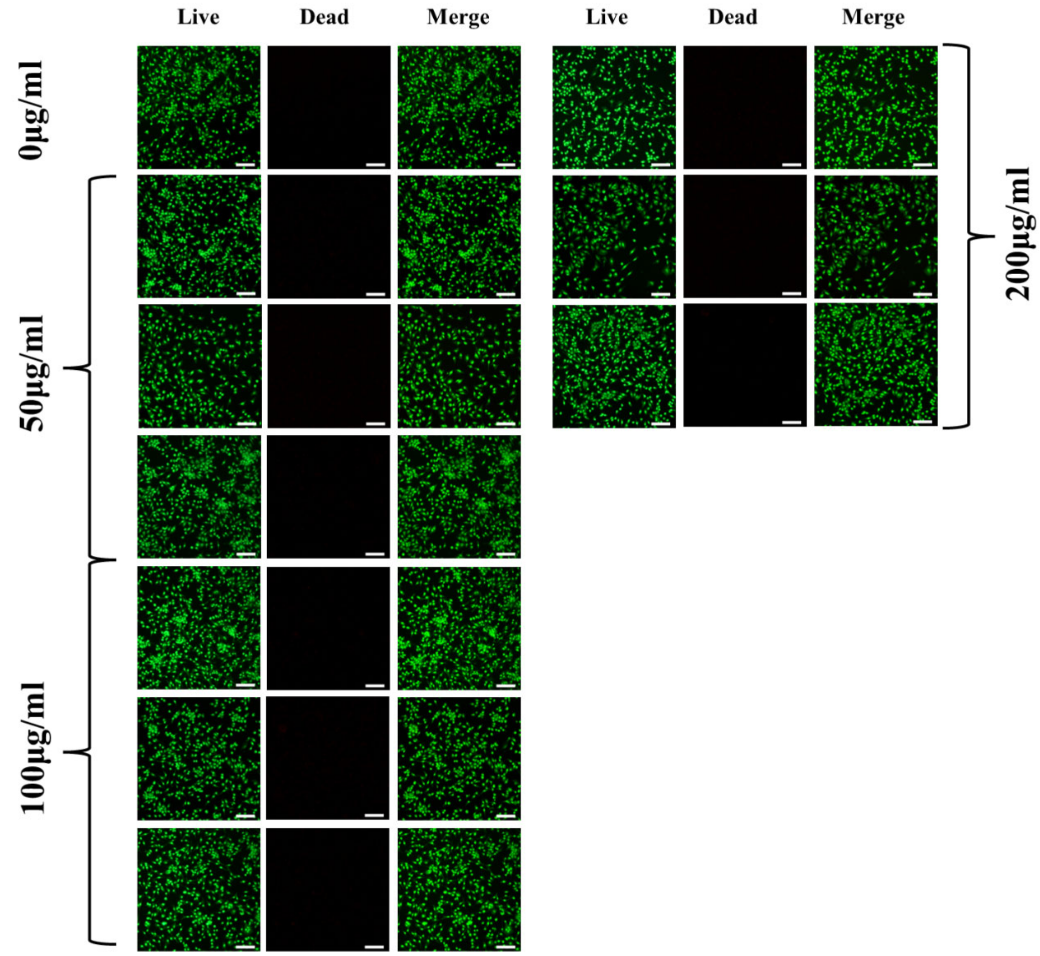


**Figure S5.** The results of the live-dead staining of cells following the co-culture of different concentrations of three nanoparticles with MC 3T3 cells are presented below. Scale bar = 100 μm. ( Except for 0 μg/ml, each concentration from top to bottom is MBG group, MBG@PDA group, MBG@PDA@Gel.)

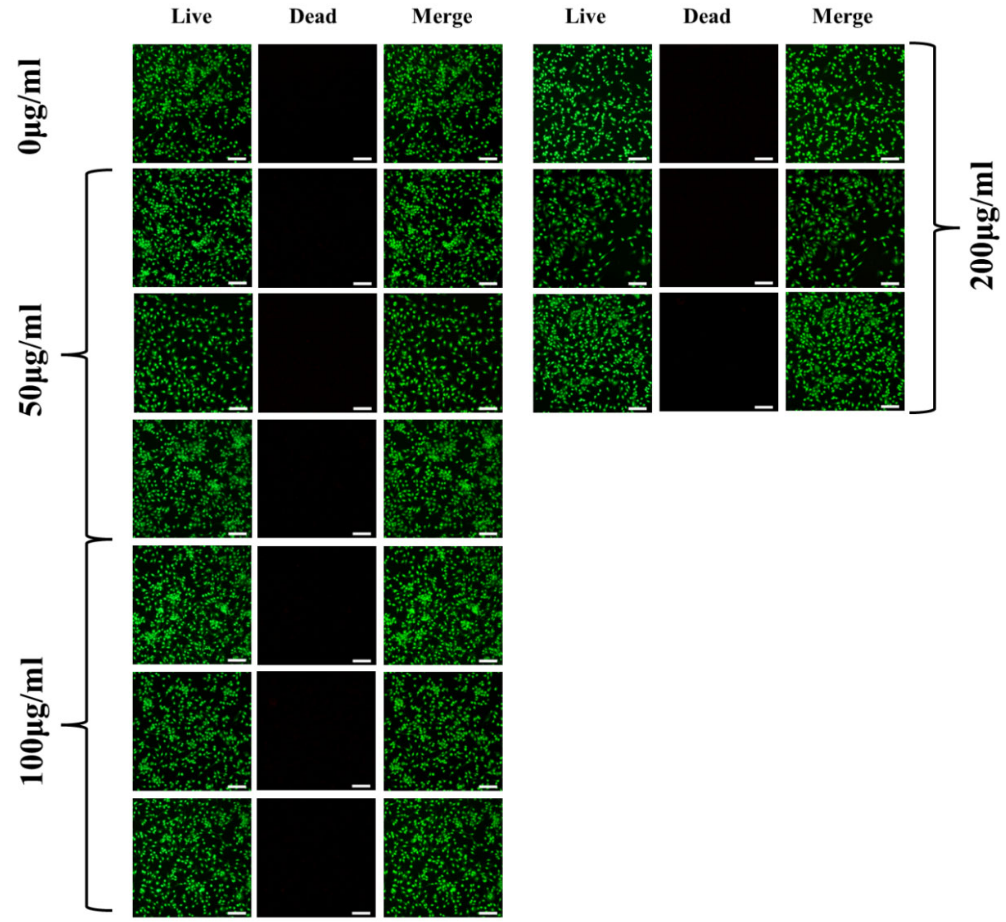


**Figure S6.**The results of the live-dead staining of cells following the co-culture of different concentrations of three nanoparticles with L929 cells are presented below. Scale bar = 100 μm. ( Except for 0 μg/ml, each concentration from top to bottom is MBG group, MBG@PDA group, MBG@PDA@Gel.)

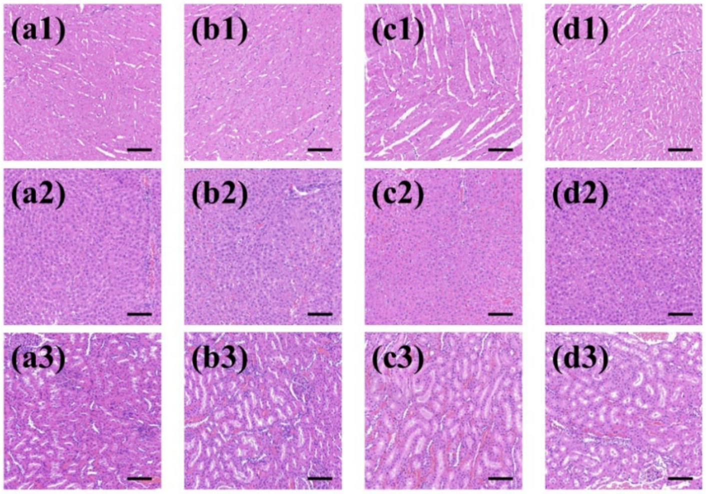


**Figure S7.** HE staining of heart, liver, and kidney in rats. (a)Control group.(b) MBG group. (c)MBG@PDA group. (d)MBG@PDA@Gel group.(a1-d1:heart,a2-d2:liver,a3-d3:kidney.)Scale bar = 100 μm.
